# The Functions and Disorders of the Reproductive Organs, in Childhood, Youth, Adult Age, and Advanced Life

**Published:** 1867-03

**Authors:** 


The Functions and Disorders of the Reproductive Organs, in Childhood, Youth, Adult Age, and Advanced Life; Considered in their Physiological, Social, and Moral Relations. By WILLIAM ACTON, M.R.C.S., late Surgeon to the Islington Dispensary, and formerly Externe to the Venerial Hospitals, Paris; Fellow of the Royal Med. and Chir. Society, etc., etc. Second American, from the Fourth London, edition. Philadelphia: LINDSAY & BLAKISTON. 1867. pp. 291. Price $3.00. For sale by S. C. GRIGGS & CO., Lake Street, Chicago.

The subjects treated in this work, are as follows:—Normal Functions in Childhood; Sexual Precocity; Masturbation in Childhood; Normal Functions in Youth; Continence; Celibacy; Early Marriages; Early Betrothals; Long Engagements; Incontinence; Masturbation in Youth; Insanity and Phthisis from Masturbation; Sexual Conditions in the Adult; Virility;

Marriage; Sexual Intercourse in Marriage; Marital Excesses; Impotence; Sexual Indifference; Erection and its Morbid Conditions; Emission and its Morbid Conditions; Semen and its Morbid Conditions; Sexual Functions and Disorders in Advanced Life; Appendix; Statistics of Marriage; Prescriptions; Exposure of Quacks. All the foregoing topics are discussed by the author with great candor and ability; and however repulsive the nature of the majority of them may be, they are all worthy of the very careful and enlightened attention of the profession. We know of no work better calculated to do good than the one now under consideration.

